# ASO Author Reflections: Discordant Clinical and Molecular Risk in Invasive Lobular Carcinoma of the Breast: The 21-Gene Recurrence Score in the National Cancer Database by Histologic Subtype

**DOI:** 10.1245/s10434-022-12104-z

**Published:** 2022-07-07

**Authors:** Mary Kathryn Abel, Rita A. Mukhtar

**Affiliations:** 1 School of Medicine, University of California San Francisco, San Francisco, CA USA; 2 Department of Surgery, University of California San Francisco, San Francisco, CA USA

## Past

The availability of molecular assays to predict the benefit of chemotherapy has allowed more tailored care for patients with breast cancer. Those with genomically low-risk tumors can be spared the potential harms of chemotherapy, whereas those with genomically high-risk tumors can be identified and receive effective treatment. However, for the subset of patients with a diagnosis of larger tumors and more nodal involvement, the clinical risk of recurrence may conflict with the predicted genomic risk of recurrence, resulting in treatment dilemmas. The authors previously showed that when the 70-gene signature is used, patients with invasive lobular carcinoma (ILC) are significantly more likely to fall into this discordant risk category than those with invasive ductal carcinoma (IDC).^1^ Indeed, 35.6% of ILC cases were found to be clinically high risk but genomically low risk compared with 19.2% of IDC cases. A high proportion of discordance means that personalizing treatment is more challenging. Recent trials have addressed this issue, with the RxPONDER trial randomizing clinically high-risk patients (those with 1–3 positive nodes) with low-risk molecular assays (21-gene recurrence score ≤ 25) to chemotherapy or no chemotherapy.^2^ Although no difference in disease-free survival was seen in the overall study population, chemotherapy was associated with improved outcomes in the pre-menopausal subset.

## Present

This analysis was performed to determine whether patients with ILC also have higher rates of clinical and genomic discordance than patients with IDC when genomic risk was assessed by the 21-gene recurrence score (RS).^3^ The study evaluated 186,867 patients with non-metastatic, hormone receptor-positive, human epidermal growth factor-2 receptor (HER2)-negative breast cancer in the National Cancer Database. The findings showed that the combination of high clinical risk and low genomic risk occurred significantly more often among patients with ILC than among those with IDC (37.8% vs. 24.9%; *p* < 0.001). This also was true for patients younger than 50 years. In all nodal categories (node-negative, 1–3 positive nodes, ≥ 3 positive nodes), the patients with ILC were significantly more likely to have low RS than the patients with IDC.

## Future

This analysis showed that patients with ILC are affected disproportionately by discordance between clinical and genomic risk, whether genomic risk is measured by the 70-gene signature (from the authors’ prior work) or by the 21-gene RS (as in this analysis). Such discordance puts patients and providers in a challenging position, with clinical stage suggesting the need for cytotoxic treatment such as chemotherapy but molecular assays suggesting limited benefit. This issue is of particular concern for patients with ILC because multiple studies have suggested decreased responsiveness to chemotherapy in the non-metastatic setting.^4,5^ The ability to tailor care for patients with ILC will require addressing the many issues that result in this discordance, including improved methods of detection to prevent diagnosis at later stages that result in lower clinical risk, ILC-specific molecular assays that can more precisely identify the subset of patients who would benefit the most from chemotherapy, and targeted therapies that have higher efficacy than those currently available.
